# Drug-Eluting Stent Restenosis: Modern Approach to a Classic Challenge

**DOI:** 10.2174/1573403X19666230103154638

**Published:** 2023-03-22

**Authors:** Teodora Donisan, Luai Madanat, Dinu V. Balanescu, Amy Mertens, Simon Dixon

**Affiliations:** 1 Department of Internal Medicine, Beaumont Hospital, Royal Oak, MI, 48073, USA;; 2 Department of Cardiovascular Medicine, Beaumont Hospital, Royal Oak, MI, 48073, USA

**Keywords:** In-stent restenosis, drug-eluting stent, bare metal stent, DES-ISR, percutaneous coronary intervention, brachytherapy

## Abstract

In-stent restenosis (ISR) is a recognized complication following percutaneous coronary intervention in which the luminal diameter is narrowed through neointimal hyperplasia and vessel remodeling. Although rates of ISR have decreased in most recent years owing to newer generation drug-eluting stents, thinner struts, and better intravascular imaging modalities, ISR remains a prevalent dilemma that proves to be challenging to manage. Several factors have been proposed to contribute to ISR formation, including mechanical stent characteristics, technical factors during the coronary intervention, and biological aspects of drug-eluting stents. Presentation of ISR can range from asymptomatic to late myocardial infarction and could be difficult to differentiate from acute thrombus formation. No definite guidelines are present on the management of ISR. In this review, we will discuss the mechanisms underlying ISR and provide insight into patient-related and procedural risk factors contributing to ISR, in addition to highlighting common treatment approaches utilized in the management of ISR.

## 1 INTRODUCTION

Recurrent in-stent restenosis (ISR) is a major cause of morbidity in patients who undergo percutaneous coronary intervention (PCI). Initial rates of restenosis with plain old balloon angioplasty (POBA) were 30-60%, with recurring lesions potentially more severe than the initial ones [1, 2]. Stenting was identified as a good option to treat restenosis after POBA [3, 4]. The introduction of drug-eluting stents (DES) led to a further reduction in restenosis rates, with superior results in terms of reintervention need, myocardial infarction, and stent thrombosis compared to BMS [5-8]. Technological advancements, such as newer antiproliferative agents, thinner struts, less polymer, and improved designs, led to a decrease in the rates of ISR with DES to as low as 5-10% in the first year after PCI [2, 7, 9, 10]. Intravascular imaging techniques, such as intravascular ultrasound (IVUS) and optical coherence tomography (OCT), have further improved PCI procedural outcomes and decreased ISR rates [11]. Nevertheless, ISR continues to occur in some patients.

The risk of DES-ISR can increase by 2% per year, and in patients with previous DES-ISR treatment failure, the recurrence risk is as high as 20% at 1 year, regardless of the approach used to treat the initial DES failure [10, 12]. DES in complex lesions (*e.g.*, long lesions, small diameter vessels, bifurcation or ostial locations, saphenous vein grafts) or in patients with a higher risk profile, the expected occurrence of ISR is more than 5-10% [13-20]. Current treatment options for DES-ISR include the use of drug-eluting balloons, high-pressure balloon angioplasty or cutting balloons, atherectomy, intravascular lithotripsy (IVL), or intravascular brachytherapy (IBT) [21-24]. In this review, we discuss the mechanisms and clinical manifestations of ISR, as well as the available data regarding treatment options for recurrent ISR.

## 2 IN-STENT RESTENOSIS

### 2.1 ISR Definition and Mechanisms of Arterial Healing after PCI

Restenosis is defined as a 50% or greater reduction in luminal diameter at the time of follow-up angiography. This binary definition of restenosis based on percentage diameter stenosis is imperfect, as it does not reflect the degree of vessel deterioration after angioplasty and the response to injury [25]. Clinical surrogates of restenosis, such as target lesion revascularization (TLR) and target vessel revascularization (TVR), although subjective, could be better indicators of local vascular deterioration.

**Fig. (1):**
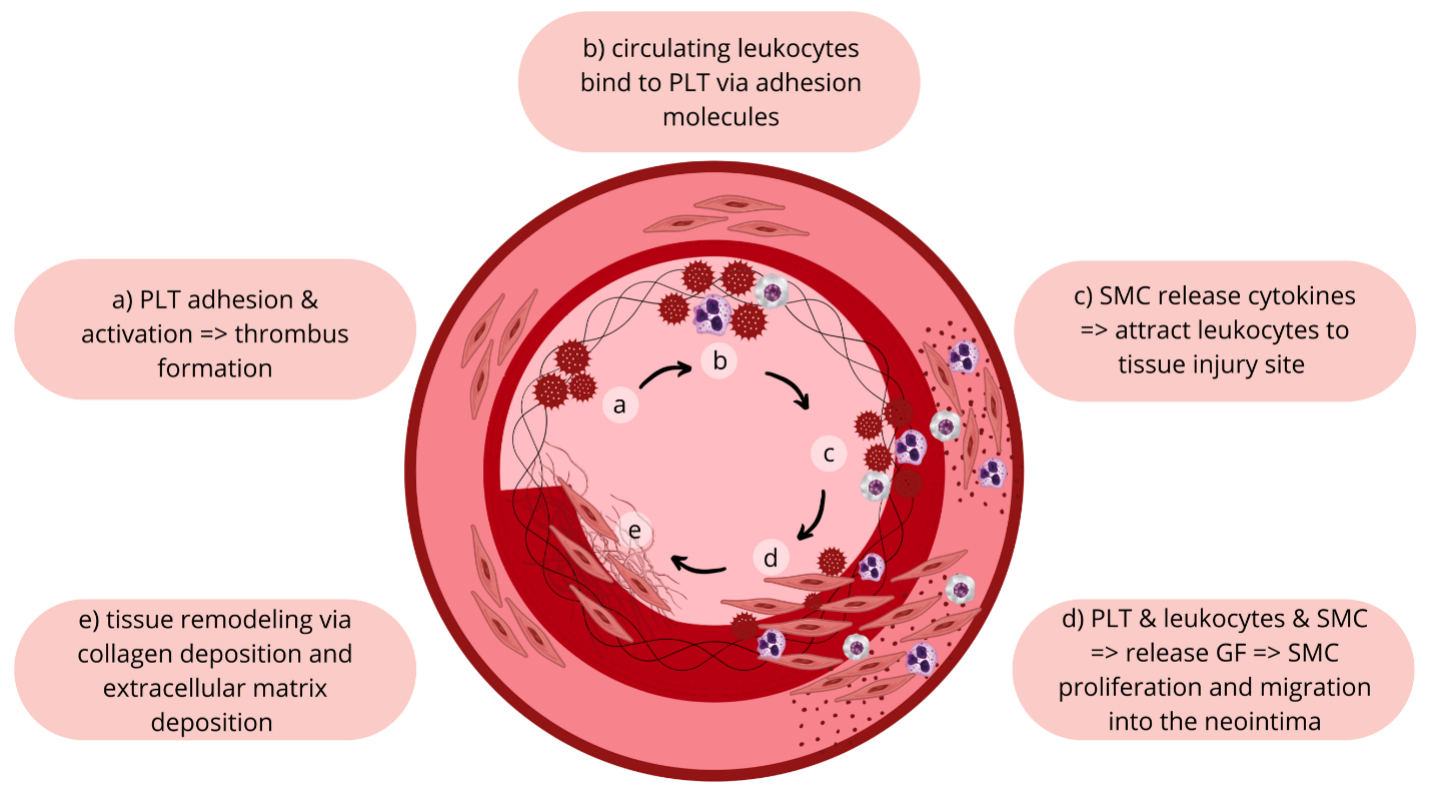
**Mechanisms for in-stent restenosis.** Endothelial damage during catheterization and stent deployment leads to endothelial activation and the production of nitrous oxide. Platelets adhere, get activated, and form a thrombus when in contact with the activated endothelium. Circulating leukocytes bind to the platelets and endothelium *via* adhesion molecules, then migrate to the tissue injury site *via* cytokines released by smooth muscle cells (SMCs) and leukocytes. Growth factors released from SMCs, platelets, and leukocytes drive the proliferation and migration of SMCs into the neointima. SMCs differentiate from contractile to secretory SMCs. Tissue remodeling occurs with the deposition of type 1 collagen and various extracellular matrix proteins. Tissue remodeling *via* matrix metall oproteinases leads to the development of in-stent restenosis. **Abbreviations:** GH, growth factors; PLT, platelets; SMC, smooth muscle cells.

The arterial wall heals after a mechanical injury caused by the percutaneous coronary intervention (PCI) through neointimal hyperplasia and vessel remodeling [25]. These processes can lead to restenosis. Animal studies have shown that neointimal hyperplasia includes multiple processes: platelet aggregation, inflammatory cell infiltration, growth factor release, smooth muscle modulation and proliferation, proteoglycan deposition, and extracellular matrix remodeling (Fig. **1**) [26-29]. Based on the complexity of these processes, antiplatelet therapies are deemed to be insufficient at preventing ISR in the BMS era, especially since inflammation appears to be at the center of the neointimal hyperplasia process.

Vascular remodeling, defined as any change in vascular dimension (either shrinkage or enlargement), is not as well understood [30]. Negative remodeling is thought to be caused by collagen synthesis by adventitial fibroblasts and tissue contraction, similar to wound healing [31, 32]. IVUS revealed the importance of negative remodeling in restenosis after POBA [33], which is virtually absent if stents are used [34]. The net superior outcomes of PCI with BMS, when compared to POBA, stem from the scaffolding properties that prevent negative remodeling, despite inducing an enhanced neointimal hyperplastic response [25]. DES are a particularly important invention in interventional cardiology, as they keep the scaffolding benefit to prevent negative vascular remodeling, while diminishing neointimal hyperplasia through the local delivery of antiproliferative medication.

### 2.2 Causes and Mechanisms of DES-ISR

DES release anti-inflammatory, immunomodulatory, and/or antiproliferative substances at the site of arterial injury. The efficiency of DES relies on its components, such as the stent platform, active pharmacologic compound, and drug carrier. Based on the novelty and performance of these components, the currently available DES can be classified into different generations: first generation (sirolimus and paclitaxel-eluting stents), second generation (everolimus, zotarolimus, umirolimus, novolimus, amphilimus, and ridaforolimus-eluting stents), and third generation (everolimus and crystalline sirolimus-eluting bioabsorbable polymer coating) [35].

Multiple factors are thought to contribute to DES-ISR, such as biological, mechanical, and technical factors (Table **1**) [36].

#### 2.2.1 Biological Factors

Patient-specific causes for DES-ISR include resistance to the antiproliferative drug on the DES (*e.g.*, due to genetic mutations) [37-39]. Some patients experience hypersensitivity reactions to the stent. These reactions are more common with BMS and first-generation DES, where the predominant stent platform material can release nickel and molybdenum, potential triggers for local contact allergies [40]. The incidence of DES hypersensitivity is unclear, but it can cause late stent thrombosis and death [41]. DES consist of multiple components (stent platform, drug, polymer carrying the drug), each one potentially causing hypersensitivity reactions. Novel DES with biodegradable polymers and improved metal alloys with lower nickel content are expected to have fewer of these complications.

The variable success of stent-based delivery of antiproliferative drugs can also be influenced by local concentrations and gradients, as mere proximity does not ensure adequate targeting [42]. Large amounts of neointimal hyperplasia and multiple stent layers could potentially decrease the diffusion of antiproliferative drugs toward the vessel media, thus minimizing the drug effect [43]. Atherosclerotic plaque content can also contribute to the neointimal formation, as neointimal thickness is found to be greater on lipid-rich lesions than on calcified lesions after second-generation DES implantation [44].

Neoatherosclerosis can develop in months to years after stent placement, whereas atherosclerosis usually develops over decades. Neoatherosclerosis has been shown to occur quicker and more frequently with DES than with BMS, and it is found in both the older generation and newer generation DES [45, 46]. The main pathogenic mechanisms proposed are the increased occurrence of uncovered struts and endothelial dysfunction, with delayed vascular healing due to antiproliferative agents on DES [46, 47]. Endothelial activation leads to increased cell permeability, leading to higher lipoprotein concentrations in the subendothelial space [48].

#### 2.2.2 Mechanical and Technical Factors

Stent underexpansion is usually caused by poor expansion during implantation. Angiography can raise suspicion of underexpansion, but it is not a good method of detecting it. Intravascular imaging, such as IVUS [49, 50] or OCT [51], can help assess correct stent expansion and positioning. Expansion is considered appropriate when the minimal stent luminal area in the stented segment is >5 mm^2^ or ≥90% of the reference luminal area [52, 53].

Stent underexpansion and malapposition can coexist but are different entities [54]. Unlike stent underexpansion, stent malapposition means that stent struts are not in contact with the vessel wall, so there is a space between the stent and the arterial intima through which blood can flow. Malapposition usually cannot be seen angiographically; intravascular imaging is the modality of choice for assessing this complication. Malapposition usually happens in cases of vessel tortuosity, complicated asymmetrical lesions, or undersized stents and could potentially predispose to stent thrombosis [55].

Stent fracture (SF) is associated with high rates of restenosis and is reported to occur in 1-8% of patients [56]. In one large multicenter trial involving 6555 patients, SF was associated with higher unadjusted rates of ISR (42.1%) compared to stents without fracture (10.7%, *P*<0.001) [57]. Several factors are thought to augment the risk of SF, including stent length (due to higher radial force and metal fatigue) [58-60], stent and balloon diameter [58, 60, 61], number of implanted stents [60], overlapping stents [62-64], and residual post stenting stenosis [58]. SF has been reported more frequently after DES implantation compared to BMS. This may be partly attributed to the fact that neointimal hyperplasia occurs more commonly after BMS implantation, masking the morphological features of stent fracture [65]. Similar to SF, a stent gap leads to interrupted coverage with DES and suboptimal local drug deposition in the vessel wall. Subsequently, the chance of developing restenosis at the gap would be higher [66].

Barotrauma outside the stented lesion could also contribute to ISR. Regions of balloon injury not covered by the exposed margins of the stent are more likely to develop restenosis [6]. This can be overcome by using shorter balloons and appropriately sized stents [36]. During complex procedures, DES polymer could be damaged, or the coating could be stripped, potentially leading to focal areas of suboptimal drug elution, contributing to increased DES-ISR risk [67, 68].

Local hemodynamic factors have also been implicated in the development of neointimal hyperplasia and restenosis in both BMS and DES [69-71]. Shear stress, defined as the tangential force derived from the friction of the flowing blood on the endothelial surface of the arterial wall, has been shown to play an intricate role in determining the amount and distribution of drug elution within the local vasculature, thus exhibiting anti-restenotic efficacy [72, 73]. Low shear stress can create a proatherogenic environment by inducing phenotypic changes in endothelial cells and regulating smooth muscle proliferation and migration to areas of endothelium post-stenting [74, 75]. Papafaklis *et al*. investigated the effect of shear stress on neointimal thickness in DES using 3D coronary artery reconstruction and blood flow computational analysis. The authors found a significant inverse relationship between the amount of neointimal hyperplasia and shear stress in paclitaxel DES. However, sirolimus elution was found to significantly attenuate the effect of shear stress on neointimal thickness [76]. Therefore, it is crucial to understand the complex relationship between local hemodynamic factors and stent mechanics in determining clinical outcomes.

### 2.3 Risk Factors for DES-ISR

#### 2.3.1 General Risk Factors

Recurrent DES-ISR is more common in patients with diabetes and chronic kidney disease and in long, calcified, and complex lesions [7, 13, 20]. Patients with diabetes and those with insulin resistance (even in the absence of diabetes) are more likely to develop ISR compared to nondiabetics [77, 78]. This is thought to be due to comorbid prothrombotic and proinflammatory conditions, such as dyslipidemia (including hypertriglyceridemia and elevated VLDL-C) and hyperuricemia [79-81]. In addition, diabetic patients tend to have more complex coronary lesions as a result of diffusely diseased small vessels [82]. The risk of ISR appears to be higher in patients with multivessel disease [78] and patients with small vessel sizes and complex lesion morphology [83].

#### 2.3.2 ISR in Venous or Arterial Bypass Grafts

Coronary artery bypass graft (CABG) is the preferred revascularization method in patients with multi-vessel coronary artery disease (CAD) unamenable to PCI. Patency rates of saphenous venous grafts (SVG) compared to arterial grafts remain poor, with occlusion rates of up to 40% within the first year and an attrition rate of 2-5% annually [84-86]. Venous grafts have the disadvantage of accelerated progression of atherosclerosis, with friable plaques, leading to high rates of graft stenosis and bypass occlusion [87, 88]. Arterial bypass grafts are less susceptible to accelerated atherosclerosis and maintain patency better than venous grafts [89]. Arterial graft dysfunction is usually caused by general atherosclerotic disease progression and neointimal hyperplasia caused by vascular trauma during CABG [90]. When comparing DES with BMS for percutaneous revascularization of internal mammary artery grafts, no statistically significant difference was found in the rates of TLR in several studies [91, 92]. Patients with a history of CABG have a higher risk of cardiac death and TVR at 5-year follow-up, even if newer generation DES are used [93]. Grafted vessels (either arterial or venous) have a significantly higher incidence of TVR than native lesions or initial graft lesions [93].

PCI is the treatment of choice for late (>1 month) graft failure [94], although it is associated with a higher incidence of MACE compared to native vessel PCI, including twice the rate of in-hospital mortality [95]. PCI in SVGs has a high risk of atheroma embolization, graft perforation, and restenosis [96]. No general consensus has been reached regarding the use of BMS *vs*. DES in SVG disease, although more recent data demonstrate an increasing trend in using DES [97]. Although DES use in venous grafts is associated with short-term lower MACE, TLR, and TVR compared to BMS, all-cause mortality and stent thrombosis are similar [98].

#### 2.3.3 ISR in Cancer

Cancer patients have multiple pathogenic mechanisms contributing to the development or worsening of CAD, including the prothrombotic and proinflammatory malignant state, the vascular toxicity of chemotherapy, and the endothelial damage caused by mediastinal radiation therapy [99]. Cancer patients have traditionally been excluded from major PCI trials, so evidence regarding PCI complications and ISR is limited to case reports [100, 101] and retrospective studies [102]. Although operators traditionally preferred the use of BMS in cancer patients because of the perceived need for shorter DAPT duration [103, 104], newer-generation DES may allow for earlier DAPT interruption and better outcomes than BMS [105-107]. In high-volume tertiary centers treating cancer patients, DES-ISR is a frequently encountered clinical challenge with suboptimal treatment methods [100, 101].

### 2.4 Clinical and Angiographic Presentation of DES-ISR

ISR can be clinically silent, but most cases present with recurrent symptoms. The presentation of DES-ISR is similar to that of BMS-ISR, with 16-66% of patients presenting with unstable angina and 1-20% with MI [108, 109]. The mean time from PCI to ISR detection is longer for DES than for BMS, with a duration of 12 months for DES [110] compared to ~5 months for BMS [111]. Late MI associated with ISR is multifactorial. It is difficult to differentiate silent occlusive restenosis from acute thrombosis, and at the same time, ISR can promote local nonocclusive thrombosis, leading to NSTEMI or unstable angina [36]. The clinical implications of ISR presenting as biomarker-positive ACS are debatable, as some studies suggest worse cardiovascular outcomes [112], whereas other research shows similar outcomes between patients presenting with ACS and patients presenting with exertional angina [113].

Mehran *et al*. described an angiographic classification of restenosis: Type 1, focal ≤ 10 mm in length; Type 2, diffuse, > 10 mm intrastent; Type 3, proliferative, > 10 mm extending beyond stent margins; and Type 4, total occlusion, restenotic lesions with TIMI flow grade of 0 [114].

DES-ISR is challenging and different from BMS-ISR. DES-ISR is focal and mostly confined to the proximal and mid-segments of the stent, rarely extending beyond stent edges, whereas BMS-ISR is usually diffuse [20, 115]. However, Bonello *et al*. found DES-ISR with a diffuse or proliferative pattern in 55% of the 122 lesions studied [116]. MI can occur more frequently and earlier after PCI in patients with diffuse ISR patterns [111].

### 2.5 Treatment Options for DES-ISR

**Fig. (2):**
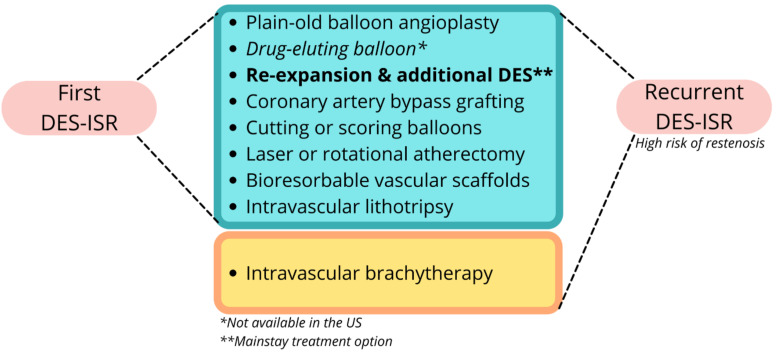
Drug-eluting stent (DES) in-stent restenosis (ISR) treatment options.

There is little evidence to guide ISR treatment. If restenosis occurred after POBA alone, initial treatment should be stenting with either BMS or DES. For BMS-ISR, the treatment is DES. For DES-ISR, traditional options include POBA, BMS, or DES (Fig. **2**) [117]. POBA may have a role in cases of mechanical stent complications, but it is associated with significant recurrence rates [118]. Drug-eluting balloons (DEB) are not currently approved for use in the US. Still, they have been proven to be more efficient than POBA for the treatment of ISR [7, 9, 10, 12-14, 117]. The usual treatment is stent re-expansion and implantation of an additional DES, with some newer-generation stents being superior to others (*e.g.*, everolimus-coated DES) [119]. The Waksman ISR classification describes ISR types and treatment suggestions based on the underlying mechanisms, including methods, such as CABG, high-pressure balloons, cutting or scoring balloons, DEB, laser or rotational atherectomy, or bioresorbable vascular scaffolds [21, 36, 120-122]. There is emerging evidence for the use of IVL for ISR as well, in combination with other treatment options [123, 124].

The aforementioned solutions are imperfect, as there is a possibility of recurrent DES-ISR, defined as ISR after a second DES or drug-eluting balloon. Even less guidance is provided regarding the treatment of recurrent DES-ISR. There is evidence suggesting that DES-ISR has higher rates of recurrence than BMS-ISR when both are treated with DES [125]. More than 15% of patients with multilayer DES will require revascularization [126]. The recurrence rates for DES-ISR are high regardless of PCI with the use of the same or different DES [18-20]. Patients with complex presentations (*i.e.*, recurrent DES-ISR with multiple failed interventions, including failed IBT) have higher MACE risks and higher rates of TVR and TLR [116].

IBT is a valid treatment option in cases of failed repeat DES or DEB implantation. Brachytherapy inhibits neointimal formation by decreasing the proliferative tissue response within the stent by delivering localized radioactive radiation to target sites of DES-ISR [122]. There is limited information regarding direct comparisons between DES and IBT use for the treatment of recurrent DES-ISR. There is promising evidence that IBT might be non-inferior to repeat DES placement, as DES-ISR recurrence rates after IBT are reported to be as high as 12% at 1 year, not different from recurrence rates after DES use [18, 43, 127].

### 2.6 What does the Future Hold?

As mentioned previously, DES-ISR is a complex multifactorial process that proves difficult to manage and has high rates of recurrence despite various treatment modalities. Ideally, intracoronary imaging with IVUS or OCT should be used to optimize the stenting procedure and avert the occurrence of ISR [128]. In addition, in cases where restenosis has occurred, intracoronary imaging has also been established as a modality in determining the etiology and characteristics of ISR and guiding the appropriate treatment plan [122, 128]. Although drug-eluting balloons (DEB) are not approved in the US, they have been given class 1 indication for the treatment of ISR by the European Society of Cardiology (ESC)/European Association for Cardio-Thoracic Surgery (EACTS) guidelines [129]. Recent studies have shown superior results with DEB compared to repeat DES implantation in the treatment of DES-ISR with higher TLR and lower complication rates at 3 years follow-up [130]. Studies have also shown promising results with the use of IBT in recurrent DES-ISR with no major safety concerns. With the lack of randomized clinical trials comparing various treatment modalities based on imaging characteristics, treatment remains to be personalized based on radiological, patient-related, and clinical factors. The use of DEB and IBT for the treatment of recurrent DES-ISR has been gaining traction in recent years and should be further investigated on a larger scale to provide sufficient data for their use.

## CONCLUSION

Recurrent ISR remains one of the most challenging conditions to treat within the spectrum of CAD, despite technological and procedural advances. The use of intravascular imaging modalities to ensure proper apposition and expansion is essential in high-risk patients with recurrent ISR. Cardiovascular mortality and morbidity remain high in patients with recurrent ISR regardless of the treatment strategy. Future directions in the treatment of DES-ISR include DEBs and IBT.

## Figures and Tables

**Table 1:** Causes for drug-eluting stent (DES) in-stent restenosis.

**Biological Factors**
Resistance to antiproliferative drug on DES (*i.e.*, genetic factors)
Hypersensitivity reactions to the stent components: o Platform (*e.g.*, nickel, molybdenum) o Drug o Polymer carrying the drug
Decreased diffusion of antiproliferative drug to vessel media can be caused by: o Neointimal hyperplasia o Multiple stent layers
Presence and type of plaque influence neointimal formation: o Lipid-rich plaque => ↑ neointimal thickness o Calcified plaque => ↓ neointimal thickness
Neoatherosclerosis formation occurs due to: o Presence of uncovered stent struts o Endothelial dysfunction => ↑ permeability => ↑ subendothelial lipoproteins
**Mechanical and Technical Factors**
Stent underexpansion o Expansion is correct if the minimal stent area is≥ 90% of the reference luminal area
Stent malapposition o There is a space between the intima and stent through which blood passes o Can occur in tortuous vessels, complicated, asymmetrical lesions, or undersized stents
Stent fracture o Risk increases with stent length, stent balloon diameter, number of implanted stents, overlapping stents, or residual stenoses
Stent gap
o Can lead to interrupted coverage with a drug-eluting stent, causing suboptimal drug deposition and increasing the risk of restenosis at the gap site
Barotrauma o Pressure on the vessels outside of the stented region can increase proliferation at stent edges
Complex procedures o Increased risk of DES polymer damage or stripped coating Hemodynamic factors o Low shear stress augments neointimal thickness

## LIST OF ABBREVIATIONS

ISR: In-Stent Restenosis
IBT: Intravascular Brachytherapy
TLR: Target Lesion Revascularization
DES: Drug-Eluting Stents
POBA: Plain Old Balloon Angioplasty
PCI: Percutaneous Coronary Intervention
TVR: Target Vessel Revascularization
